# Relationship Between Non-Alcoholic Fatty Liver Disease and Degree of Hepatic Steatosis and Bone Mineral Density

**DOI:** 10.3389/fendo.2022.857110

**Published:** 2022-03-14

**Authors:** Ruijie Xie, Mingjiang Liu

**Affiliations:** Department of Hand Surgery, The Affiliated Nanhua Hospital, Hengyang Medical School, University of South China, Hengyang, China

**Keywords:** bone mineral density, osteoporosis, NHANES, non-alcoholic fatty liver disease, cross-sectional study, hepatic steatosis

## Abstract

**Background:**

The liver and bones are both active endocrine organs that carry out several metabolic functions. However, the link between non-alcoholic fatty liver disease (NAFLD) and bone mineral density (BMD) is still controversial. The goal of this study was to discover if there was a link between non-alcoholic fatty liver disease and bone mineral density in US persons aged 20 to 59 years of different genders and races.

**Methods:**

Using data from the National Health and Nutrition Examination Survey (NHANES) 2017–2018, multivariate logistic regression models were utilized to investigate the association between NAFLD and lumbar BMD. Fitted smoothing curves and generalized additive models were also used.

**Results:**

The analysis included a total of 1980 adults. After controlling for various variables, we discovered that NAFLD was negatively linked with lumbar BMD. The favorable connection of NAFLD with lumbar BMD was maintained in subgroup analyses stratified by sex, race and age in men, other race and aged 20-29 years. The relationship between NAFLD and lumbar BMD in blacks and people aged 40-49 years was a U-shaped curve with the inflection point: at 236dB/m and 262dB/m. Furthermore, we discovered that liver advanced fibrosis and liver cirrhosis were independently connected with higher BMD, while no significant differences were detected in severe liver steatosis and BMD.

**Conclusions:**

Our study found an independently unfavorable relationship between NAFLD and BMD in persons aged 20 to 59. We also discovered a positive link between BMD and advanced fibrosis and cirrhosis. More research is needed to back up the findings of this study and to look into the underlying issues.

## Background

Osteoporosis is a long-term disorder marked by reduced bone mineral density (BMD) that affects a huge number of people (1). According to the International Osteoporosis Foundation, more than 30 percent of women and more than 20 percent of men over the age of 50 have osteoporosis or osteopenia, putting them at risk for osteoporotic fractures (2). Simultaneously, the prevalence of osteoporosis continues to climb as the population ages and expands (3). Apart from genetics, age, and gender, other variables that affect bone metabolisms, such as food intake and lifestyle, have lately received a lot of attention (4–6). Meanwhile, scientists are working to discover novel ways to prevent and treat osteoporosis.

NAFLD (non-alcoholic fatty liver disease) is the most common chronic liver disease and one of the leading causes of severe liver disease across the world. In the absence of severe alcohol consumption or secondary reasons, NAFLD is characterized as excessive fat infiltration into the liver. Currently, the prevalence in Asia is about one out of four people with NAFLD, which is comparable to many Western countries (7). In addition, a physically inactive lifestyle and a rising trend of metabolic diseases such as hypertension, type 2 diabetes, dyslipidemia and obesity are associated with the prevalence and development of NAFLD (8).

Both the bone and the liver are active endocrine organs with a variety of metabolic functions (9, 10). A growing body of research implies a relationship between NAFLD and low BMD (11–14). According to various studies, patients with NAFLD are more likely to have low BMD and an increased risk of osteoporotic fractures, and the underlying mechanism is convoluted and unknown (11). The occurrence of significant liver fibrosis as determined by vibration controlled and transient elastography (VCTE) was connected to poor BMD in NAFLD in a small number of studies (15). The link between low BMD and NAFLD has only been studied in a few large-scale longitudinal investigations. Furthermore, the mechanism underlying this is unknown, but Circulating molecules, insulin resistance, TNF-α and vitamin D insufficiency appear to be potential linkages (16). As a result, we assessed the connection of NAFLD with BMD in adults in this study using a comprehensive fraction of individuals aged 20 to 59 from the National Health and Nutrition Examination Survey (NHANES).

## Materials and Methods

### Data Source and Study Population

**Figure 1:**
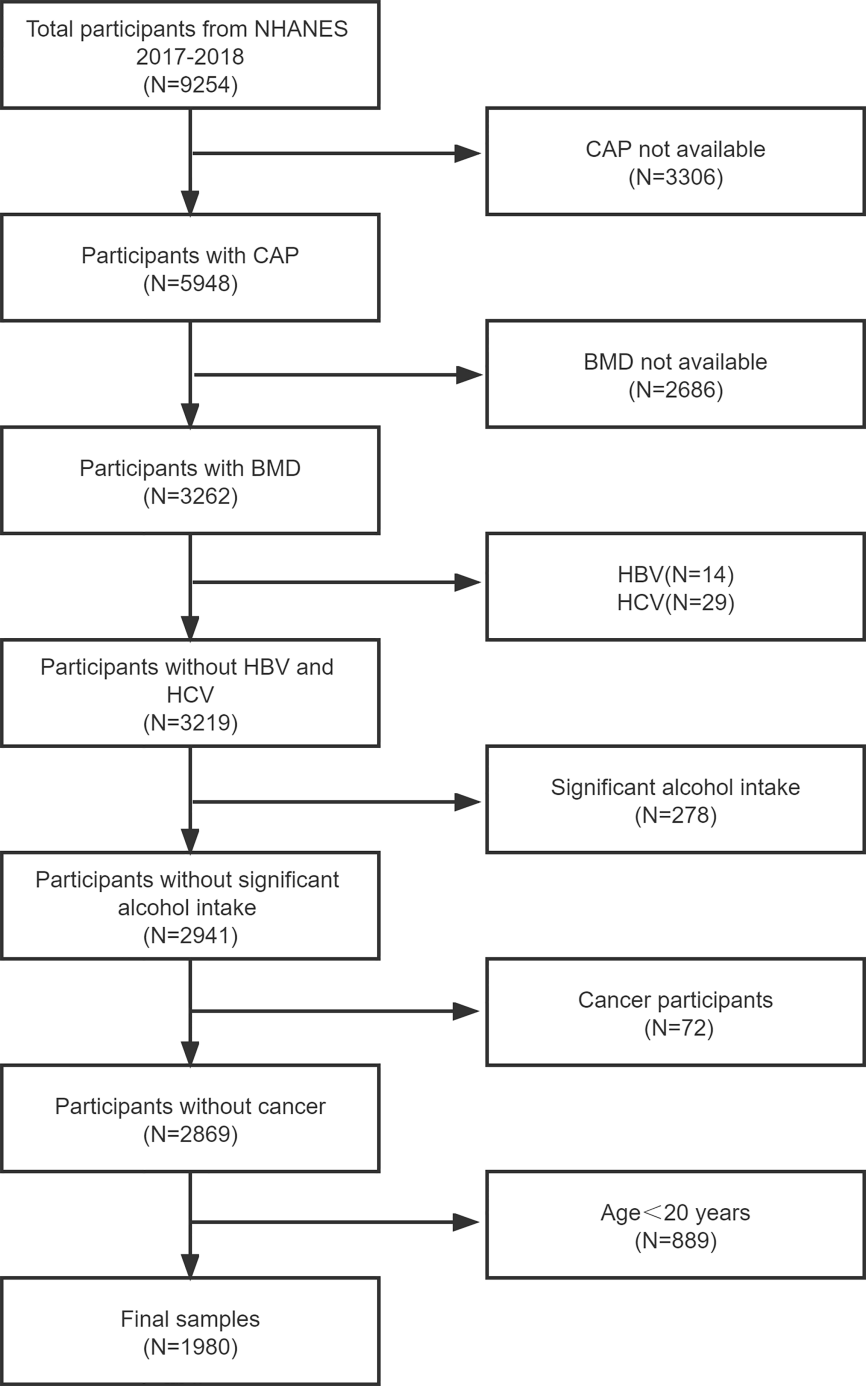
Flow chart of participants selection. NHANES, National Health and Nutrition Examination Survey; CAP, controlled attenuation parameter; BMD, bone mineral density; hepatitis B virus, HBV; hepatitis C virus, HCV.

The NHANES is a major, continuing cross-sectional survey in the United States that aims to give objective statistics on health issues and address emerging public health concerns among the general public. The NHANES datasets were utilized for this investigation from 2017 to 2018. The participants in the research had to be between the ages of 20 and 59. Among the 1980 eligible adults, we excluded 3306 individuals with missing Median CAP data, 2686 with missing BMD data, 752 participants with significant alcohol consumption, 889 individuals younger than 20 years, 14 hepatitis B antigen-positive and 29 hepatitis C antibody-positive or hepatitis C RNA-positive samples, and 72 individuals with cancer diagnoses. Finally, 1980 people were enrolled in the study. Finally, 1980 people were enrolled in the study (**Figure 1**).

### Ethics Statement

The National Center for Health Statistics Research Ethics Review Board authorized the protocols for the NHANES and got signed informed consent. After anonymization, the NHANES data is available to the public. This enables academics to transform data into a study-able format. We agree to follow the study’s data usage guidelines to guarantee that data is only utilized for statistical analysis and that all experiments are carried out in compliance with applicable standards and regulations.

### Study Variables

Clinicians use VCTE as a noninvasive approach to determine the prevalence and severity of NAFLD in clinical practice, and it has been found to be trustworthy. NHANES staff used FibroScan^®^ model 502 V2 Touch equipped to conduct VCTE evaluations on participants throughout the 2017-2018 period. According to a recent landmark study, controlled attenuation parameter values, which also be called CAP, ≥274 dB/m was considered suggestive of NAFLD status since had 90% sensitivity in detecting all degrees of liver steatosis (17). Dual-energy X-ray absorptiometry was performed using a Hologic QDR 4500A device and Apex software version 3.2 by qualified radiology technologists to assess lumbar BMD. Covariates in multivariate models may cause the correlations between urinary caffeine and caffeine metabolites and lumbar BMD to be muddled. Age, gender, race, body mass index, poverty to income ratio, education, diabetes status, waist circumference, Glycated hemoglobin, Total cholesterol, Triglyceride, LDL- cholesterol, HDL- cholesterol, ALT, ALP, GGT, AST, Serum creatinine, Serum iron, Lumbar bone mineral density, CAP and LSM were all covariates in this study. The NHANES website (https://www.cdc.gov/nchs/nhanes/) has a thorough explanation of how these variables are calculated.

### Statistical Analysis

We used R (http://www.r-project.org) and EmpowerStats (http://www.empowerstats.com) for all statistical analyses, with statistical significance set at P < 0.05. Because the goal of NHANES is to produce data that is representative of the civilian noninstitutionalized population in the United States, all estimates were calculated using sample weights in accordance with NCHS’s analytical guidelines. Model 1 had no variables adjusted, model 2 had age, gender, and race adjusted, and model 3 had all of the covariates listed in **Table 1** adjusted. There were also subgroup analyses performed. A weighted generalized additive model and smooth curve fitting were employed to deal with non-linearity.

**Table 1:** Weighted characteristics of the study population based on CAP.

	Non-NAFLD	NAFLD	Severe steatosis	*P* value
	(CAP<274, n = 1210)	(274≤CAP<302, n = 281)	(CAP≥302, n = 489)	
Age (years)	36.835 ± 11.891	40.665 ± 11.886	41.183 ± 10.901	<0.00001
*Gender (%)*				<0.00001
Male	44.600	53.731	61.260	
Female	55.400	46.269	38.740	
*Race/Ethnicity (%)*				<0.00001
Non-Hispanic White	57.645	48.903	56.806	
Non- Hispanic Black	13.689	11.455	9.606	
Mexican American	7.567	15.343	14.303	
Other Race	21.099	24.300	19.285	
*Diabetes (%)*				<0.00001
Yes	1.735	5.032	11.909	
No	98.265	94.968	88.091	
Moderate activities				0.09520
Yes	52.879	49.436	49.004	
No	47.121	50.564	50.996	
Smoke at least 100 cigarettes				0.30105
Yes	32.692	33.267	33.097	
No	67.308	66.733	66.903	
Broken or fractured a hip				0.54585
Yes	0.327	1.328	0.368	
No	99.673	98.672	99.632	
Broken or fractured a wrist				0.03451
Yes	17.919	11.184	8.346	
No	82.081	88.816	91.654	
Broken or fractured spine				0.73514
Yes	4.079	3.601	1.686	
No	95.921	96.399	98.314	
Ever taken prednisone or cortisone daily				0.39680
Yes	10.170	8.125	5.263	
No	89.830	91.875	94.737	
Income to poverty ratio	3.063 ± 1.659	3.069 ± 1.601	2.995 ± 1.591	0.74727
BMI (Kg/m2)	26.484 ± 5.634	30.883 ± 5.703	34.975 ± 7.195	<0.00001
Waist circumference (cm)	90.845 ± 14.221	102.653 ± 12.657	112.635 ± 15.418	<0.00001
Laboratory features				
HbA1c (%)	5.327 ± 0.552	5.624 ± 0.907	5.842 ± 1.061	<0.00001
Total cholesterol (mmol/L)	4.753 ± 0.953	4.965 ± 0.965	5.049 ± 0.935	<0.00001
Triglyceride(mmol/L)	1.015 ± 0.658	1.647 ± 1.629	1.921 ± 1.709	<0.00001
LDL- cholesterol(mmol/L)	2.787 ± 0.853	2.976 ± 0.909	3.055 ± 0.833	0.00022
HDL- cholesterol(mmol/L)	1.467 ± 0.382	1.270 ± 0.367	1.190 ± 0.305	<0.00001
ALT (IU/L)	19.855 ± 13.137	26.308 ± 17.980	31.708 ± 22.117	<0.00001
AST (IU/L)	21.072 ± 11.305	21.365 ± 8.724	24.567 ± 13.545	<0.00001
ALP(IU/L)	71.010 ± 22.289	75.605 ± 19.544	78.627 ± 21.957	<0.00001
GGT (IU/L)	23.071 ± 26.912	31.363 ± 30.740	37.902 ± 34.435	<0.00001
Serum creatinine (umol/L)	75.129 ± 17.899	75.437 ± 20.164	75.237 ± 18.139	0.77658
Serum iron(umol/L)	17.028 ± 7.614	15.618 ± 5.559	15.196 ± 5.879	<0.00001
CAP (dB/m)	217.329 ± 36.446	287.384 ± 7.872	341.624 ± 30.199	<0.00001
LSM (kPa)	4.676 ± 1.981	6.121 ± 6.987	7.500 ± 8.264	<0.00001
Lumbar bone mineral density (g/cm^2^)	1.058 ± 0.151	1.034 ± 0.137	1.039 ± 0.150	0.00935

Mean+SD for continuous variables: P value was calculated by weighted linear regression model.

% for Categorical variables: P value was calculated by weighted chi-square test.

## Results

### Baseline Characteristics

The demographic and laboratory data of the participants (1210 Non-NAFLD, 281 NAFLD and 489 Severe steatosis) are presented in **Table 1**. Compared to Non-NAFLD participants, NAFLD participants and Severe steatosis participants were more likely to be male, Mexican American, and diabetic populations. Participants with NAFLD and Severe steatosis had significantly higher BMI, waist circumference and higher wrist fractured rate, and significantly higher levels of Glycated hemoglobin, Total cholesterol, Triglyceride, LDL- cholesterol, ALT, AST, ALP, GGT, CAP, and LSM, while HDL- cholesterol, and Serum iron, and Lumbar bone mineral density were lower. The weighted characteristics of the study population based on LSM are shown in **Table S1**.

### Relationship Between NAFLD and BMD

**Figure 2:**
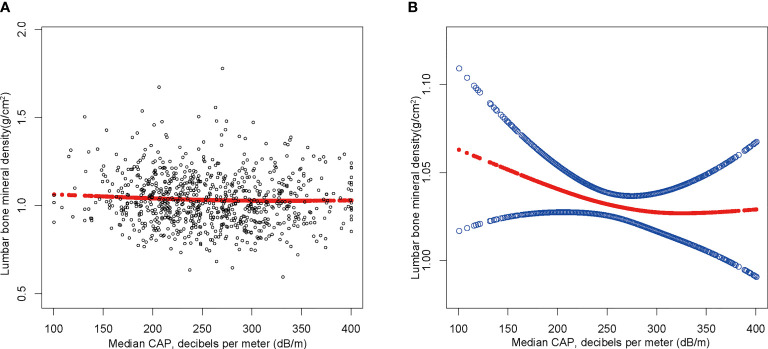
The association between NAFLD and lumbar bone mineral density. **(A)** Each black point represents a sample. **(B)** The solid red line represents the smooth curve fit between variables. Blue bands represent the 95% of confidence interval from the fit. Age, gender, race, body mass index, poverty to income ratio, education, diabetes status, waist circumference, Glycated hemoglobin, Total cholesterol, Triglyceride, LDL- cholesterol, HDL- cholesterol, ALT, ALP, GGT, AST, Serum creatinine, Serum iron, Lumbar bone mineral density, CAP and LSM were adjusted.

The findings of the multivariate regression analysis are shown in **Table 2** and **Figure 2**. NAFLD was negatively linked with lumbar BMD in the unadjusted model [-0.022 (-0.035, -0.008)]. However, this significant correlation becomes insignificant after adjusting for the covariates in Model 2[-0.012 (-0.026, 0.001)] and Model 3[-0.013 (-0.049, 0.023)]. With the point of inflection discovered by two-piecewise linear regression model, at 367(dB/m) (**Table 5**).

**Table 2:** Association between NAFLD and lumbar bone mineral density (g/cm2) stratified by gender.

	Model 1:β(95*%* CI), *p*	Model 2:β(95*%* CI), *p*	Model 3:β(95*%* CI), *p*
Non-NAFLD	Reference	Reference	Reference
NAFLD	-0.022 (-0.035, -0.008) 0.00206	-0.012 (-0.026, 0.001) 0.07913	-0.039 (-0.081, 0.003) 0.07250
Males			
Non-NAFLD	Reference	Reference	Reference
NAFLD	-0.029 (-0.048, -0.009) 0.00495	-0.020 (-0.040, 0.000) 0.05423	-0.021 (-0.050, 0.062) 0.08322
Females			
Non-NAFLD	Reference	Reference	Reference
NAFLD	-0.014 (-0.033, 0.005) 0.15842	-0.003 (-0.022, 0.016) 0.72778	-0.017 (-0.052, 0.039) 0.06461

Model 1: No covariates were adjusted. Model 2: Age, gender, race were adjusted.

Model 3: Age, gender, race, body mass index, poverty to income ratio, education, smoking behavior, Moderate activities, Diabetes status, Waist circumference, HbA1c (%), Total cholesterol, Triglyceride, LDL- cholesterol, HDL- cholesterol, ALT, ALP, GGT, AST, Serum creatinine, Serum iron, Lumbar bone mineral density, CAP and LSM were adjusted.

*In the subgroup analysis stratified by gender or race, the model is not adjusted for the stratification variable itself.

### Subgroup Analysis

After adjusting for covariates, the results of subgroup analysis, smooth curve fittings and generalized additive models showed that the association among NAFLD and BMD was mainly present in males, other race and participants aged 20 to 29. Detailed information on the subgroup analysis is shown in **Tables 2**–**4**.

**Table 3:** Association between NAFLD and lumbar bone mineral density (g/cm2) stratified by race.

Race/Ethnicity (%)	Model 1:β(95*%* CI), *p*	Model 2:β(95*%* CI), *p*	Model 3:β(95*%* CI), *p*
Non-Hispanic White			
Non-NAFLD	Reference	Reference	Reference
NAFLD	-0.013 (-0.039, 0.013) 0.32101	-0.008 (-0.035, 0.018) 0.54638	-0.016 (-0.082, 0.042) 0.51292
Non- Hispanic Black			
Non-NAFLD	Reference	Reference	Reference
NAFLD	-0.005 (-0.039, 0.030) 0.77792	0.003 (-0.033, 0.038)	0.030 (-0.078, 0.118)
		0.88626	0.46515
Mexican American			
Non-NAFLD	Reference	Reference	Reference
NAFLD	-0.021 (-0.049, 0.006) 0.12916	-0.021 (-0.049, 0.007) 0.14525	-0.041 (-0.128, 0.011) 0.31224
Other Race			
Non-NAFLD	Reference	Reference	Reference
NAFLD	-0.024 (-0.046, -0.003) 0.02556	-0.012 (-0.026, 0.001) 0.07913	-0.011 (-0.090, 0.068) 0.78868

Model 1: No covariates were adjusted. Model 2: Age, gender, race were adjusted.

Model 3: Age, gender, race, body mass index, poverty to income ratio, education, smoking behavior, Moderate activities, Diabetes status, Waist circumference, HbA1c (%), Total cholesterol, Triglyceride, LDL- cholesterol, HDL- cholesterol, ALT, ALP, GGT, AST, Serum creatinine, Serum iron, Lumbar bone mineral density, CAP and LSM were adjusted.

*In the subgroup analysis stratified by gender or race, the model is not adjusted for the stratification variable itself.

**Table 4:** Association between NAFLD and lumbar bone mineral density (g/cm2) stratified by age.

Age	Model 1:β(95*%* CI), *p*	Model 2:β(95*%* CI), *p*	Model 3:β(95*%* CI), *p*
Age (20-29)			
Non-NAFLD	Reference	Reference	Reference
NAFLD	-0.064 (-0.089, -0.038) <0.00001	-0.050 (-0.076, -0.025) 0.00013	-0.051 (-0.139, 0.011) 0.05521
Age (30-39)			
Non-NAFLD	Reference	Reference	Reference
NAFLD	-0.017 (-0.041, 0.007) 0.17505	-0.010 (-0.034, 0.015) 0.44259	-0.028 (-0.078, 0.032) 0.63545
Age (40-49)			
Non-NAFLD	Reference	Reference	Reference
NAFLD	-0.008 (-0.037, 0.020) 0.56467	-0.001 (-0.029, 0.027) 0.94431	0.020 (-0.055, 0.109) 0.62401
Age (50-59)			
Non-NAFLD	Reference	Reference	Reference
NAFLD	0.011 (-0.020, 0.042)	0.004 (-0.028, 0.035)	0.029 (-0.048, 0.107)
	0.49400	0.82339	0.45933

Model 1: No covariates were adjusted. Model 2: Age, gender, race were adjusted.

Model 3: Age, gender, race, body mass index, poverty to income ratio, education, smoking behavior, Moderate activities, Diabetes status, Waist circumference, HbA1c (%), Total cholesterol, Triglyceride, LDL- cholesterol, HDL- cholesterol, ALT, ALP, GGT, AST, Serum creatinine, Serum iron, Lumbar bone mineral density, CAP and LSM were adjusted.

*In the subgroup analysis stratified by gender or race, the model is not adjusted for the stratification variable itself.

**Figure 3:**
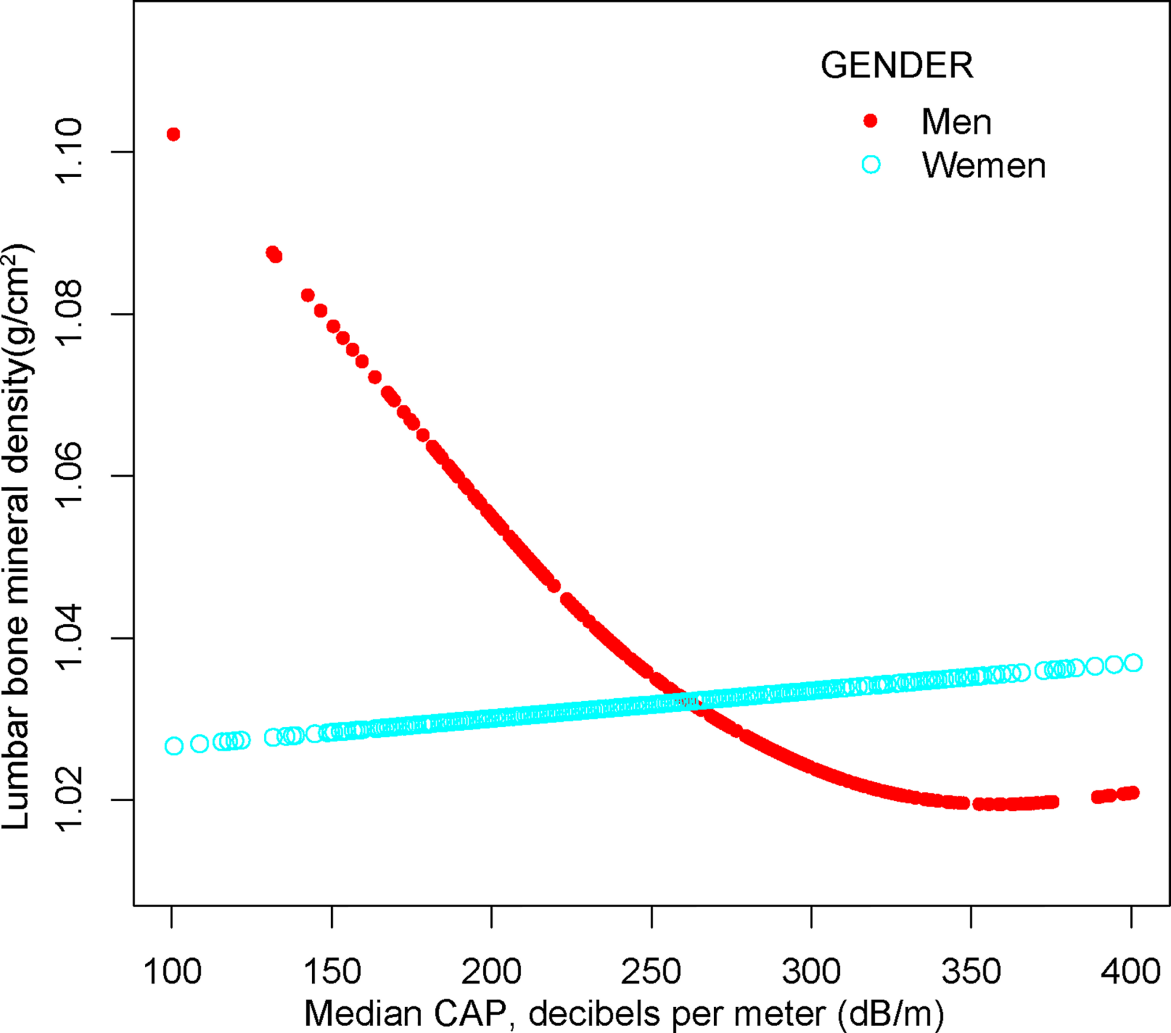
The association between NAFLD and lumbar bone mineral density stratified by gender. Age, gender, race, body mass index, poverty to income ratio, education, diabetes status, waist circumference, Glycated hemoglobin, Total cholesterol, Triglyceride, LDL- cholesterol, HDL- cholesterol, ALT, ALP, GGT, AST, Serum creatinine, Serum iron, Lumbar bone mineral density, CAP and LSM were adjusted.

For males, NAFLD exhibited a significant inverse association with BMD in Model 1[-0.029 (-0.048, -0.009)], but not in Model 2[-0.020 (-0.040, 0.000)] and Model3[-0.004 (-0.060, 0.052)]. In addition, the nonlinear relationship was characterized by smooth curve fittings and generalized additive models (**Table 2** and **Figure 3**).

**Figure 4:**
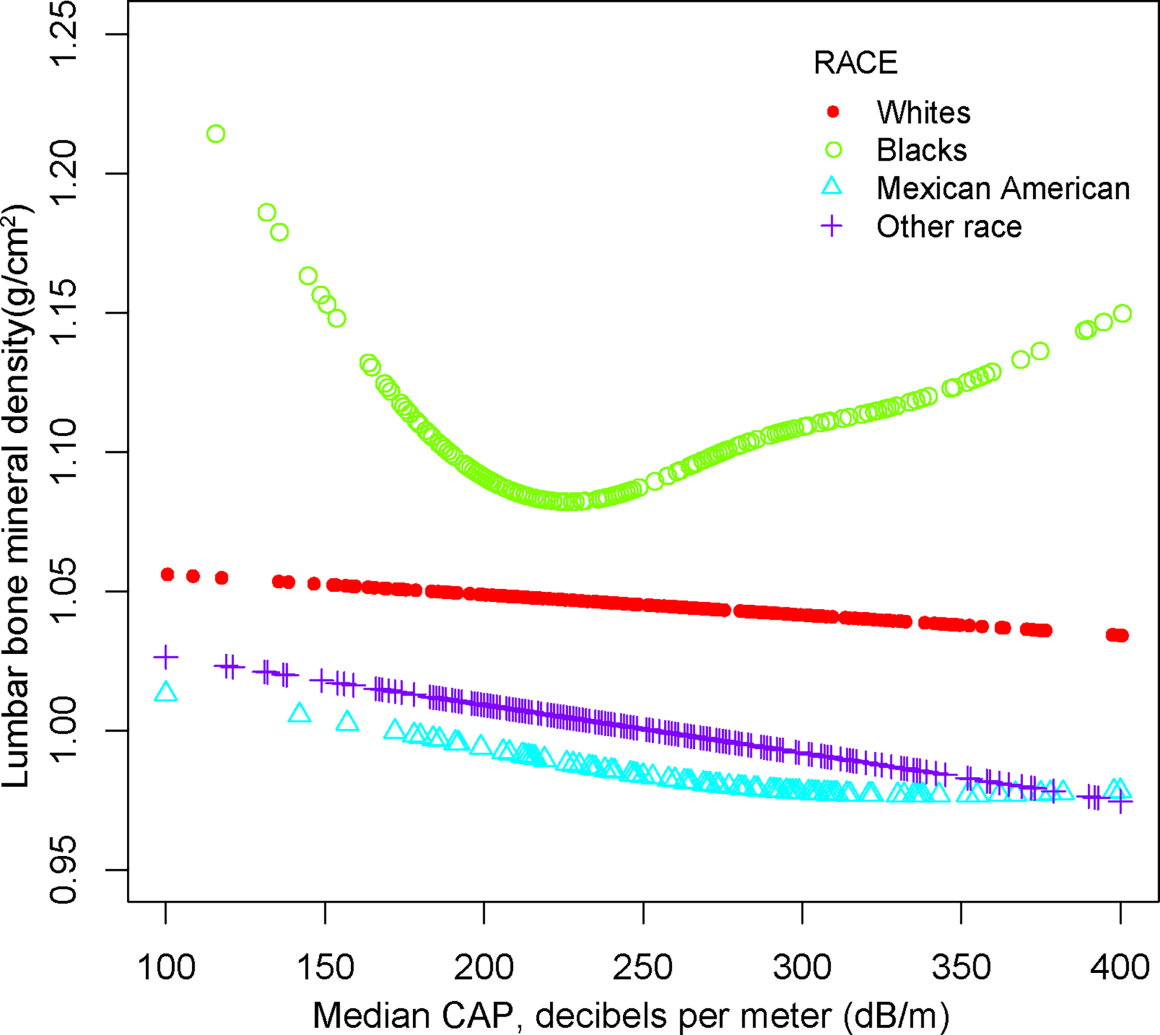
The association between NAFLD and lumbar bone mineral density stratified by race. Age, gender, race, body mass index, poverty to income ratio, education, diabetes status, waist circumference, Glycated hemoglobin, Total cholesterol, Triglyceride, LDL- cholesterol, HDL- cholesterol, ALT, ALP, GGT, AST, Serum creatinine, Serum iron, Lumbar bone mineral density, CAP and LSM were adjusted.

For other race, the adverse association as same as males in Model 1[-0.024 (-0.046, -0.003)], but not in Model 2[-0.012 (-0.026, 0.001)] and Model3[-0.013 (-0.049, 0.023)]. Of note, when stratified by race, we found a U-shape relationship between NAFLD and BMD in blacks (**Table 3** and **Figure 4**). With the point of inflection discovered by two-piecewise linear regression model, at 236(dB/m) (**Table 5**).

**Table 5:** Threshold effect analysis of NAFLD on lumbar bone mineral density using two-piecewise linear regression model.

Lumbar bone mineral density	Adjusted β(95％CI)
	*P* value
***NAFLD***	
Inflection point	367
CAP<236(dB/m)	-0.000 (-0.000, 0.000)
	0.7497
CAP>236(dB/m)	0.001 (-0.001, 0.003)
	0.3573
Log likelihood ratio	0.342
***Non-Hispanic black***	
Inflection point	236
CAP<236(dB/m)	-0.005 (-0.008, -0.001)
	0.0101
CAP>236(dB/m)	-0.000 (-0.001, 0.001)
	0.8620
Log likelihood ratio	0.009
***Aged 40-49***	
Inflection point	262
CAP<262(dB/m)	0.000 (-0.000, 0.001)
	0.8448
CAP>262(dB/m)	-0.004 (-0.006, -0.001)
	0.0015
Log likelihood ratio	0.028

Age, gender, race, body mass index, poverty to income ratio, education, smoking behavior, Moderate activities, Diabetes status, Waist circumference, HbA1c (%), Total cholesterol, Triglyceride, LDL- cholesterol, HDL- cholesterol, ALT, ALP, GGT, AST, Serum creatinine, Serum iron, Lumbar bone mineral density, CAP and LSM were adjusted.

**Figure 5:**
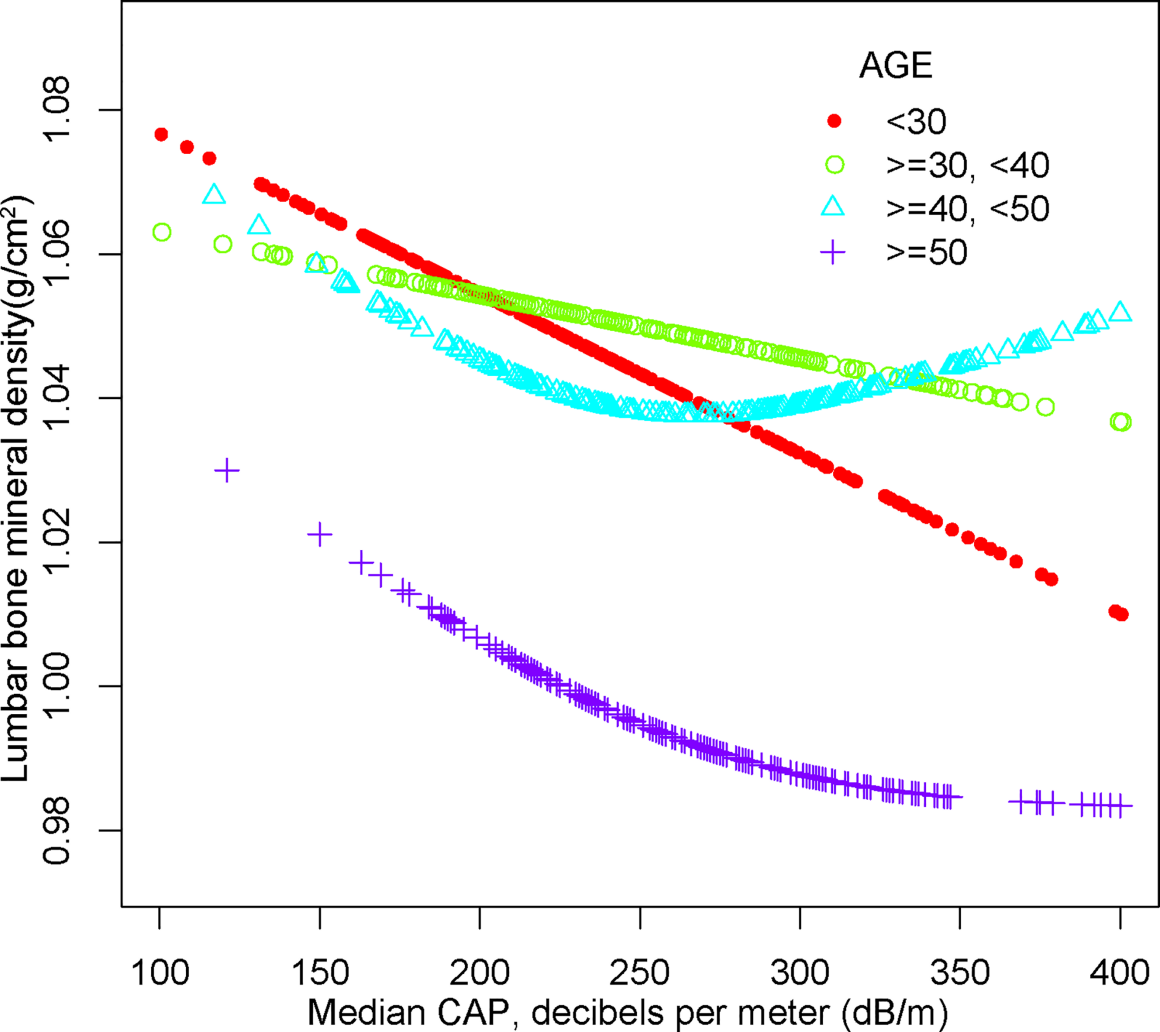
The association between NAFLD and lumbar bone mineral density stratified by age. Age, gender, race, body mass index, poverty to income ratio, education, diabetes status, waist circumference, Glycated hemoglobin, Total cholesterol, Triglyceride, LDL- cholesterol, HDL- cholesterol, ALT, ALP, GGT, AST, Serum creatinine, Serum iron, Lumbar bone mineral density, CAP and LSM were adjusted.

For people aged 20-29, there is a significant negative association with NAFLD and BMD in Model 1[-0.064 (-0.089, -0.038)], Model 2[-0.050 (-0.076, -0.025)] but not in Model3[-0.058 (-0.119, 0.003)]. Furthermore, we found a U-shape relationship between NAFLD and BMD in people aged 40-49 years, when stratified by age in **Table 4** and **Figure 5**. With the point of inflection identified using a two-piecewise linear regression model, at 262(dB/m) (**Table 5**).

### Relationship Between Degree of Hepatic Steatosis and BMD

We further investigated the connection among degree of hepatic steatosis and BMD in adults with NAFLD, we found a significant positive association between advanced liver fibrosis and BMD in Model1[-0.064 (-0.089, -0.038)], Model2[-0.064 (-0.089, -0.038)] but not in Model3[-0.064 (-0.089, -0.038)]. And there is a significant positive association between liver cirrhosis and BMD in Model1[0.067 (0.021, 0.112)], Model2[0.068 (0.024, 0.112)] and Model3[0.153 (0.032, 0.274)]. However, no significant differences were found in severe liver steatosis with BMD as well as Significant liver fibrosis with BMD. **Table 6** provide more details on the subgroup analysis.

**Table 6:** Association between degree of hepatic steatosis and lumbar bone mineral density (g/cm2).

Exposure	Model 1:β(95*%* CI), *p*	Model 2:β(95*%* CI), *p*	Model 3:β(95*%* CI), *p*
Severe steatosis			
CAP<302	Reference	Reference	Reference
CAP≥302 (n = 489)	-0.015 (-0.030, 0.001) 0.05991	-0.008 (-0.023, 0.008) 0.33794	-0.020 (-0.051, 0.020) 0.35510
Significant fibrosis			
LSM<8.0	Reference	Reference	Reference
LSM≥8.0 (n = 59)	0.011 (-0.015, 0.037)	0.013 (-0.013, 0.039)	-0.013 (-0.065, 0.046) 0.78231
	0.40881	0.31822	
Advanced fibrosis			
LSM<9.7	Reference	Reference	Reference
LSM≥9.7 (n = 39)	0.049 (0.014, 0.084)	0.051 (0.017, 0.086)	0.059 (-0.054, 0.101)
	0.00631	0.00367	0.13638
Cirrhosis			
LSM<13.6	Reference	Reference	Reference
LSM≥13.6 (n = 33)	0.067 (0.021, 0.112)	0.068 (0.024, 0.112)	0.150 (0.031, 0.264)
	0.00387	0.00256	0.02010

Model 1: No covariates were adjusted. Model 2: Age, gender, race were adjusted.

Model 3: Age, gender, race, body mass index, poverty to income ratio, education, smoking behavior, Moderate activities, Diabetes status, Waist circumference, HbA1c (%), Total cholesterol, Triglyceride, LDL- cholesterol, HDL- cholesterol, ALT, ALP, GGT, AST, Serum creatinine, Serum iron, Lumbar bone mineral density, CAP and LSM were adjusted.

*In the subgroup analysis stratified by gender or race, the model is not adjusted for the stratification variable itself.

## Discussion

In this study of individuals aged 20-59 years, we demonstrated the negative association between NAFLD and BMD. In addition, on subgroup analysis, however, we discovered a U-shaped relationship among other studies of NAFLD and BMD in other races and people aged 20-29. Moreover, based on the non-invasive fibrosis markers, we found a positive correlation between BMD and Advanced fibrosis and Cirrhosis.

Clinical studies on the relationship between NAFLD and BMD in adults are still inconclusive. And the majority of these epidemiological studies are centered on Asian and menopausal female populations, with only a handful focusing on European and American males. There was no notable change in BMD among patients with NAFLD and controls, according to a recent meta-analysis of five cross-sectional studies (18). NAFLD was likewise linked to self-reported osteoporotic fractures in the other meta-analysis, but not to poor BMD (14). Other studies, on the other hand, refuted this conclusion. The findings of cohort research involving 4318 Chinese with NAFLD and 17,272 Chinese without NAFLD revealed that NAFLD may enhance the risk of new-onset osteoporosis (19). A Korean cross-sectional study of 3739 premenopausal women discovered a negative link between NAFLD and BMD (12). Other Korean and Chinese cross-sectional investigations backed up the same conclusion (20–24), as well as a cohort study from America (25). NAFLD was strongly connected to an increased risk of low BMD in men but not in women, and in other race but not in whites, blacks, or Mexican Americans, according to our findings. According to previous studies, NAFLD is a hermaphroditic dimorphic condition that is more frequent in males and postmenopausal women, whereas inadequate bone mineral density is more frequent in postmenopausal women (26, 27). However, there are few studies on racial differences in NAFLD and BMD and further epidemiological studies based on racial stratification analysis are needed to clarify the causes.

Clinical investigations on the link between steatosis severity and BMD are scarce and controversial. Kim et al. discovered that substantial liver fibrosis as measured by hepatic transient elastography is independently linked with low BMD in a cross-sectional study of 231 asymptomatic Korean participants (15). A new study in NAFLDs looked at the relationship between liver fibrosis and BMD (28). They discovered that NAFLD-related hepatic fibrosis was linked to lower BMD in postmenopausal women with T2DM or IGR. According to a remarkable study (17), severe steatosis defined as CAP ≥ 302, advanced fibrosis defined as LSM ≥ 9.7 kPa, and cirrhosis defined as LSM ≥ 13.6 kPa were noticed. We investigated the association between steatosis severity and BMD by this definition (29). In contrast to previous findings, we discovered that liver advanced fibrosis and liver cirrhosis were independently connected with higher BMD, while no significant differences were detected in severe liver steatosis and BMD.

The mechanisms behind the relationship between NAFLD and BMD are unclear. There are various probable causes for this phenomenon, according to relevant studies. NAFLD can worsen insulin resistance and trigger the production of a slew of pro-inflammatory cytokines and bone-influencing molecules, all of which can contribute to bone demineralization and osteoporosis (30, 31). In addition to this, there is growing evidence that NAFLD causes alterations in the production of several molecular coordinators that may be detrimental to bone health, such as overproduction of TNF-a (32) and deficiencies in vitamin D (33), osteopontin (34) and osteoprotegerin (35). Moreover, circulating molecules may have different effects on bone metabolism by affecting early childhood obesity (36) or the progression of NAFLD (37). However, it is reasonable to believe that increased body weight is a prevalent trait of people with NAFLD (38), may help to prevent bone loss by increasing mechanical loads and improving cortical bone growth. Observations in people with obesity or type 2 diabetes are similar (39). Long-term fracture risk in patients with NAFLD may be underestimated by BMD values alone. It is conceivable to assume, based on the findings of this study, that NAFLD may have a sex-related differential influence on fracture risk. However, given that an increased risk of self-reported osteoporotic fractures among patients with NAFLD has only been seen in two cross-sectional studies conducted in China, it is still unclear if these findings can be generalized to other ethnic communities (22, 23). Furthermore, sex hormone levels and body fat deposition might be plausible causes for the discrepancies between men and women. In postmenopausal women, estrogen insufficiency is believed to be the leading cause of low bone mineral density (40, 41). Estrogen works to retain bone mass by reducing bone resorption by regulating osteoclast activity through the estrogen receptor (42, 43). NAFLD and the effect of estrogen insufficiency in women may contribute to the development of low BMD in an additive or synergistic manner. More research is needed to properly understand the function of NAFLD in the development of bone loss, taking into account the fact that various effects exist depending on gender. However, we feel that further prospective studies and mechanistic research are needed to better understand this crucial subject, particularly in non-Asian populations.

Most cohort and cross-sectional research have focused on postmenopausal women and Asians to yet. Little is known regarding the relationship between NAFLD and BMD in non-Asian, younger populations. Our findings are extremely relevant to the entire population since we used a nationally representative sample. We were also able to undertake subgroup analyses of NAFLD and lumbar spine BMD across gender and ethnicity, and evaluate the relationship between the degree of hepatic steatosis and bone mineral density, thanks to our large sample size. However, it is crucial to acknowledge the study’s limitations. First, our study’s cross-sectional design makes it hard to conclude a causal association between NAFLD and lumbar BMD in adults. To understand the specific mechanism of the relationship between NAFLD and BMD, further fundamental mechanistic research and large sample prospective studies are required. Second, NAFLD was diagnosed based on vibration controlled and transient elastography, which may have understated the prevalence of the disease. Third, the part of missing data from the NHANES database 2017-2018 on the usage of medication, history of fracture that can alter BMD could have skewed the results. Fourth, due to the limitations of the NHANES database, we were unable to obtain data on T score or Z score, which could also affect our assessment of the participants’ osteoporosis.

## Conclusion

Our study found an independently unfavorable relationship between NAFLD and lumbar BMD in persons aged 20 to 59. This connection followed a U-shaped pattern among blacks and persons aged 40-49 years. We also discovered a positive link between BMD and advanced fibrosis and cirrhosis. Our findings may provide insight into prospective osteoporosis preventative and treatment approaches. More high-quality prospective studies are needed to corroborate or refute our findings on this research issue, as well as a more in-depth analysis of gender and ethnic disparities.

## Data Availability Statement

Publicly available datasets were analyzed in this study. This data can be found here: www.cdc.gov/nchs/nhanes/.

## Ethics Statement

Written informed consent was obtained from the individual(s) for the publication of any potentially identifiable images or data included in this article.

## Author Contributions

RX and ML designed the research. RX collected, analyzed the data, and drafted the manuscript. RX and ML revised the manuscript. All authors contributed to the article and approved the submitted version.

## Funding

This study Funded by the Scientific Research Project of Hunan Health and Family Planning Commission to ML (A2017018).

## Conflict of Interest

The authors declare that the research was conducted in the absence of any commercial or financial relationships that could be construed as a potential conflict of interest.

## Publisher’s Note

All claims expressed in this article are solely those of the authors and do not necessarily represent those of their affiliated organizations, or those of the publisher, the editors and the reviewers. Any product that may be evaluated in this article, or claim that may be made by its manufacturer, is not guaranteed or endorsed by the publisher.

## Abbreviations

NAFLD, Non-alcoholic fatty liver disease; BMD, bone mineral density; NHANES, National Health and Nutrition Examination Survey; VCTE, vibration controlled and transient elastography; CAP, controlled attenuation parameter; HBV, hepatitis B virus; HCV, hepatitis C virus; BMI, body mass index.
