# Supplementary material for: Relationship Between Non-Alcoholic Fatty Liver Disease and Degree of Hepatic Steatosis and Bone Mineral Density
Source: Front Endocrinol (Lausanne). 2022 Mar 14;13:857110. doi: 10.3389/fendo.2022.857110 (PMC8964007; doi:10.3389/fendo.2022.857110)
Supplement: Supplementary file 1 [file Table_1.pdf]

Table1: Weighted characteristics of the study population based on LSM

	**Normal group (LSM<8.0, n = 1849)**	**Significant fibrosis (8.0≤LSM<9.7, n = 59)**	**Advanced fibrosis(9.7≤ LSM<13.6, n = 39)**	**Cirrhosis(LSM ≥13.6, n = 33)**	***P* value**
Age (years)	38.071 ± 11.880	40.694 ± 10.733	44.305 ± 11.047	43.678 ± 9.037	0.00022
Gender (%)					0.12895
Male	49.378	50.442	71.865	49.732	
Female	50.622	49.558	28.135	50.268	
Race/Ethnicity (%)					0.52099
Non-Hispanic White	55.658	67.754	51.633	69.813	
Non- Hispanic Black	12.552	7.963	16.857	10.863	
Mexican American	10.426	6.330	10.440	5.304	
Other Race	21.364	17.953	21.070	14.020	
Diabetes (%)					<0.00001
Yes	3.730	15.354	26.480	10.379	
No	96.290	84.646	73.520	89.621	
Broken or fractured a hip					0.97555
Yes	0.559	100.000	100.000	100.000	
No	99.441	0.000	0.000	0.000	
Broken or fractured a wrist					0.13142
Yes	15.354	3.194	100.000	100.000	
No	84.646	96.806	0.000	0.000	
Broken or fractured spine					0.97887
Yes	3.361	1.963	7.796	100.000	
No	96.639	98.037	92.204	0.000	
Ever taken prednisone or cortisone daily					0.00646
Yes	8.257	100.000	100.000	41.221	
No	91.743	0.000	0.000	58.779	
Income to poverty ratio	3.041 ± 1.635	2.987 ± 1.658	3.022 ± 1.674	3.463 ± 1.516	0.49944
BMI (Kg/m2)	28.484 ± 6.260	31.884 ± 9.943	37.385 ± 7.971	44.539 ± 9.824	<0.00001
Waist circumference (cm)	96.106 ± 15.513	105.241 ± 22.925	119.223 ± 18.343	133.204 ± 17.322	<0.00001
Laboratory features					
HbA1c (%)	5.451 ± 0.725	6.146 ± 1.539	5.849 ± 1.052	5.983 ± 0.968	<0.00001
Total cholesterol (mmol/L)	4.851 ± 0.958	4.639 ± 0.946	4.933 ± 1.119	5.133 ± 0.840	0.08478
LDL-cholesterol(mmol/L)	2.873 ± 0.862	2.551 ± 0.818	2.824 ± 1.057	3.299 ± 0.482	0.08390

Table1: Weighted characteristics of the study population based on LSM

HDL-cholesterol(mmol/L)	1.384 ± 0.382	1.331 ± 0.306	1.228 ± 0.517	1.113 ± 0.287	0.00001
ALT (IU/L)	22.863 ± 15.991	30.302 ± 18.488	44.605 ± 41.953	30.082 ± 22.422	<0.00001
AST (IU/L)	21.594 ± 10.964	25.485 ± 11.856	32.412 ± 27.893	25.481 ± 17.371	<0.00001
ALP(IU/L)	72.796 ± 21.622	86.127 ± 27.940	76.253 ± 24.251	81.597 ± 24.664	<0.00001
GGT (IU/L)	26.864 ± 26.870	32.569 ± 30.892	62.605 ± 103.083	35.423 ± 37.252	<0.00001
Serum iron(umol/L)	16.328 ± 6.682	20.269 ± 14.019	17.149 ± 6.324	13.617 ± 4.883	0.00002
CAP (dB/m)	251.786 ± 59.452	273.758 ± 67.535	337.374 ± 58.126	354.222 ± 49.348	<0.00001
LSM (kPa)	4.731 ± 1.191	8.604 ± 0.548	11.126 ± 0.973	31.758 ± 20.208	<0.00001
Lumbar bone mineral density (g/cm^2)	1.050 ± 0.149	1.019 ± 0.144	1.071 ± 0.151	1.116 ± 0.155	0.00906

Mean+SD for continuous variables: P value was calculated by weighted linear regression model.

% for Categorical variables: P value was calculated by weighted chi-square test.
